# The Impact of Mandibular Anatomy on the Incidence of Bad Splits in Sagittal Split Osteotomy: A Retrospective Study

**DOI:** 10.3390/jcm15145661

**Published:** 2026-07-19

**Authors:** Tuncer Akdogan, Huseyin Can Tukel

**Affiliations:** Department of Oral & Maxillofacial Surgery, Faculty of Dentistry, Cukurova University, Balcalı Campus, Adana 01330, Turkey; cantukel@gmail.com

**Keywords:** osteotomy, sagittal split ramus, cone-beam computed tomography, orthognathic surgical procedures, intraoperative complications

## Abstract

**Objectives**: The objective of this study was to conduct three-dimensional cone-beam computed tomography (CBCT) analysis of posterior mandibular anatomy in order to investigate its potential correlation with the occurrence of bad splits during sagittal split osteotomy (SSO). **Methods**: A retrospective review was conducted on 56 patients (112 hemimandibles) who underwent bilateral SSO between 2016 and 2022. Distances between the mandibular canal and the borders of the mandible, along with anatomical landmarks, were measured. Sites with and without bad splits were compared using chi-square and Mann–Whitney U tests (*p* = 0.05). **Results**: The overall incidence of bad split was 8.9% per site (10/112) and 14.3% per patient (8/56). Patient-related variables, including age, sex, skeletal deformity type (Class II vs. Class III), and the presence of impacted third molars, were not significantly associated with bad split (all *p* > 0.05). Among the morphometric parameters, the distance from the mandibular canal to the inferior cortical border at the mesial of the second molar (M2inferior) was significantly greater in the bad-split group (*p* = 0.04). Other measurements, including ramal thickness, anteroposterior length, and canal position relative to buccal and inferior cortices, were not significantly different. Reliability analysis demonstrated good to excellent agreement for all measurements. A greater inferior mandibular border thickness at the mesial of the second molar appears to increase the risk of a bad split during SSO. **Conclusions**: Routine preoperative CBCT evaluation and careful attention to inferior border osteotomy in this region may help reduce complications. Further prospective, standardised studies are needed to confirm these findings.

## 1. Introduction

Sagittal split osteotomy (SSO) of the mandible is a commonly performed procedure for the correction of dentofacial deformities. Owing to its versatility and predictable outcomes, SSO has become the standard technique for correcting mandibular deformities. Nevertheless, despite continuous refinements in surgical techniques, fixation methods, and preoperative planning, intraoperative and postoperative complications—including bleeding, infection, hardware failure, inferior alveolar nerve injury, condylar malposition, and bad splits—remain clinically relevant [1].

A bad split refers to unfavorable or irregular fractures of the mandible that occur during SSO, and it is probably the most unwanted complication that may lead to other complications if not addressed properly [2]. Although relatively uncommon, it remains one of the most challenging intraoperative complications because it may compromise fixation, prolong operative time, and adversely affect postoperative healing and stability. Various studies have reported that the incidence of bad splits ranges from 1% to 9.2% [3,4]. A bad split can result in infection, delayed healing, malunion, relapse, malocclusion and temporomandibular disorders [5,6]. Many studies have suggested that bad splits can result from various factors, such as a lack of surgical experience, the surgical technique, the age of the patient, the presence of impacted third molars, and some anatomical deviations such as a high-riding lingula and a narrow ramus [7,8]. To minimise the risk of bad splits, several technical modifications and refinements to the conventional sagittal split osteotomy have been proposed, including alterations in osteotomy design, splitting techniques, and the use of piezoelectric surgery. Despite these advances, unfavorable fractures continue to occur, suggesting that patient-specific anatomical characteristics may also play a crucial role in determining fracture patterns [9,10,11]. However, there is a paucity of literature regarding the anatomical factors of the mandible that may lead to bad splits, and most often, the three-dimensional anatomy of the mandible in relation to SSO is overlooked [3].

Thus, the objective of this study was to conduct a three-dimensional cone-beam computed tomography (CBCT) analysis of mandibular anatomy related to SSO, with regard to potential correlations with bad splits.

## 2. Materials and Methods

### 2.1. Patient Selection

This retrospective study was conducted in the Department of Oral and Maxillofacial Surgery, Cukurova University, and included patients who underwent SSO between January 2016 and February 2022. A total of 138 patients who underwent bilateral sagittal split osteotomy (SSO) between January 2016 and February 2022 were retrospectively screened. Among these patients, 56 patients with available preoperative and postoperative CBCT scans were included in the study. Ethical approval was obtained from the Clinical Research Ethics Committee of Cukurova University (Meeting No. 125, dated 16 September 2022). All participants were informed about the study, and written informed consent was obtained prior to data collection. The participants were categorised by sex assigned at birth, following SAGER (Sex and Gender Equity in Research) guidelines, which recommend clear definitions for sex and gender classifications. Only binary categories of male and female were included, as no participants identified as otherwise. This approach aligns with SAGER guidelines, which recommend explaining the rationale for selected sex categories. Among the 56 patients, 36 (64.3%) were female, and 20 (35.7%) were male.

Inclusion criteria:

- Patients who underwent bilateral sagittal split osteotomy between 2016 and 2022.
- Availability of complete preoperative and postoperative CBCT scans.
- Complete clinical records.

Exclusion criteria:

- Previous mandibular surgery.
- Craniofacial syndromes.
- Mandibular pathology or trauma.
- Inadequate CBCT image quality.
- Missing clinical records.

Because postoperative CBCT is not routinely performed after orthognathic surgery at our institution, only patients with available preoperative and postoperative CBCT scans were eligible for inclusion. Consequently, all operated hemimandibles included in the analysis underwent postoperative CBCT evaluation, allowing consistent assessment of unfavorable fractures (bad splits). This inclusion criterion may have introduced sampling bias and should be considered when interpreting the reported incidence of bad splits.

### 2.2. Surgical Procedure

The sagittal split osteotomy procedures for individuals who agreed to participate in this study were performed by three different senior surgeons following the same standardised surgical protocol. The sagittal split osteotomy procedure was performed under general anaesthesia using the techniques described by Trauner and Obwegeser [12] and modified by Hunsuck and Dal Pont [13]. The modified SSO technique, derived from the classic method of Trauner and Obwegeser, includes Dal Pont’s anteriorly positioned vertical osteotomy and Epker’s complete osteotomy of the inferior mandibular cortex. This approach was utilised to increase the surface area between bone segments, thereby optimising rigid fixation and promoting enhanced postoperative healing.

### 2.3. Acquisition of CBCT Images

The CBCT records for all patients were obtained at the Faculty of Dentistry, Cukurova University. The records were obtained using a CBCT device (Planmeca Promax^®^ 3D Mid, Helsinki, Finland). The tomography device operates at 90 kV and 10 mA. It performs a 360° rotation around the patient, scanning a field of view (FOV) of 450 mm × 450 mm × 436 mm in approximately 27 s with an average slice thickness of 0.5 mm. The acquired data were stored in the Digital Imaging and Communications in Medicine (DICOM) format. The patient’s DICOM data were transferred to the Planmeca Promax^®^ 3D Mid (version 3.8.1.R; Planmeca Oy, Helsinki, Finland) software for analysis. During image acquisition, patients were positioned using the manufacturer’s head stabilisation system with laser-guided alignment, with the Frankfort horizontal plane oriented parallel to the floor and the facial midline aligned with the vertical reference beam. Before measurements, all CBCT datasets were reviewed and reoriented to correct minor variations in head position. The Frankfort horizontal plane was aligned parallel to the horizontal reference, while the midsagittal plane was aligned perpendicular to the horizontal reference in the axial view. All linear measurements were subsequently performed on the standardised reoriented images.

### 2.4. Measurements Evaluated on CBCT Images

The landmarks and reference planes used in this study were established by utilising various sources [14,15].

**Figure 1:**
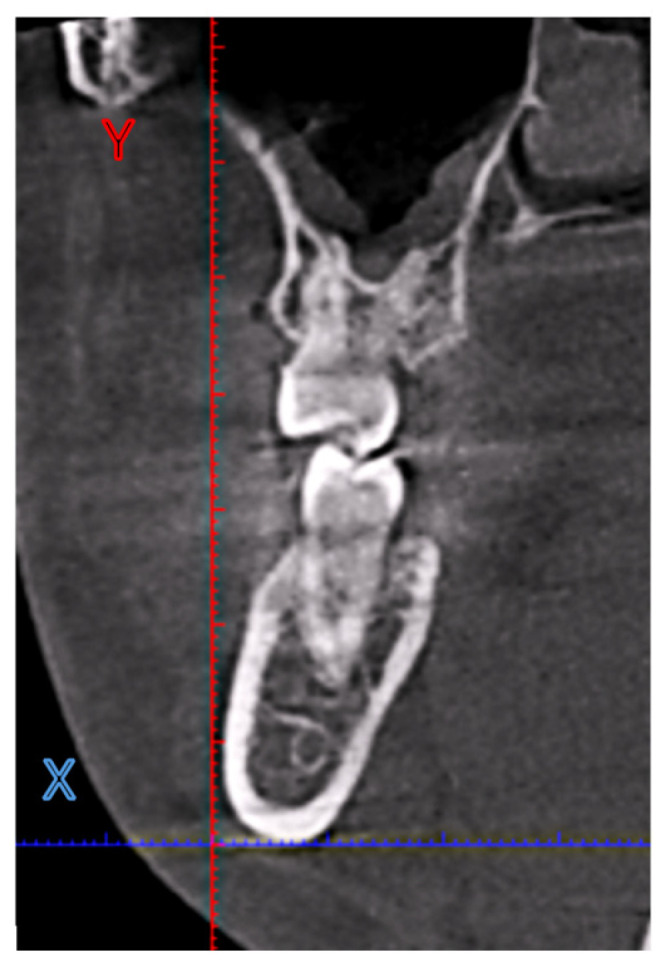
Y and X planes.

- Y Plane: A vertical plane that is perpendicular to the ground (Figure 1).
- X Plane: A horizontal plane that is parallel to the ground (Figure 1).
- Mandibular Foramen: An anatomical structure located just above the centre of the inner surface of the mandibular ramus, surrounded by bone at the beginning of the mandibular canal.
- Mandibular Sigmoid Notch: The deepest point of the concavity extending between the mandibular condyle and coronoid process.
- Mandibular Lingula: A bony prominence located on the medial side of the mandibular ramus above the mandibular foramen.

### 2.5. Length Measurements

**Figure 2:**
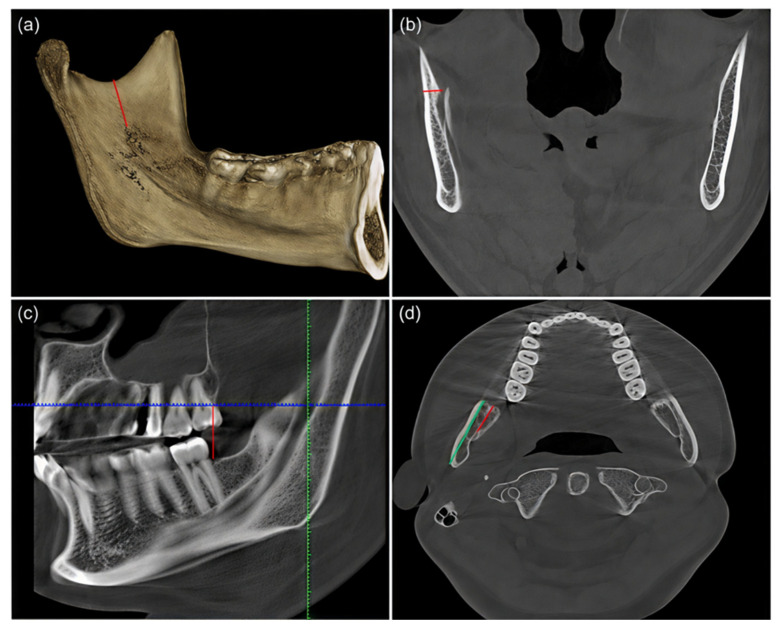
(**a**) Length between the lingula and the sigmoid notch in a 3D model. (**b**) Coronal section illustration of the mediolateral width of the ramus at the level of the lingula. (**c**) CBCT illustration of the vertical distance from the mandibular foramen to the distal region of the mandibular second molar (Green line: Coronal axis, Blue line: Axial axis). (**d**) Axial section illustration of the anteroposterior length of the ramus at the level of the mandibular foramen (green line) and the distance from the mandibular foramen to the anterior border of the ramus (red line).

- LintoSig: Distance from the lingula of the mandible to the sigmoid notch (Figure 2a).
- Rammedlat: Mediolateral thickness of the ramus at the level of the lingula of the mandible (Figure 2b).
- M2toFor: Vertical distance from the distal of the mandibular second molar to the mandibular foramen (Figure 2c).
- RanttoFor: Distance from the mandibular foramen to the anterior border of the ramus (Figure 2d).
- RanttoPost: Anteroposterior length of the ramus at the level of the mandibular foramen (Figure 2d).

For all molar-level measurements, standardised coronal sections were selected according to reproducible dental landmarks: the contact point between the mandibular second premolar and first molar (M1), the contact point between the mandibular first and second molars (M2), and the coronal section passing through the distal surface of the mandibular second molar (M3).

**Figure 3:**
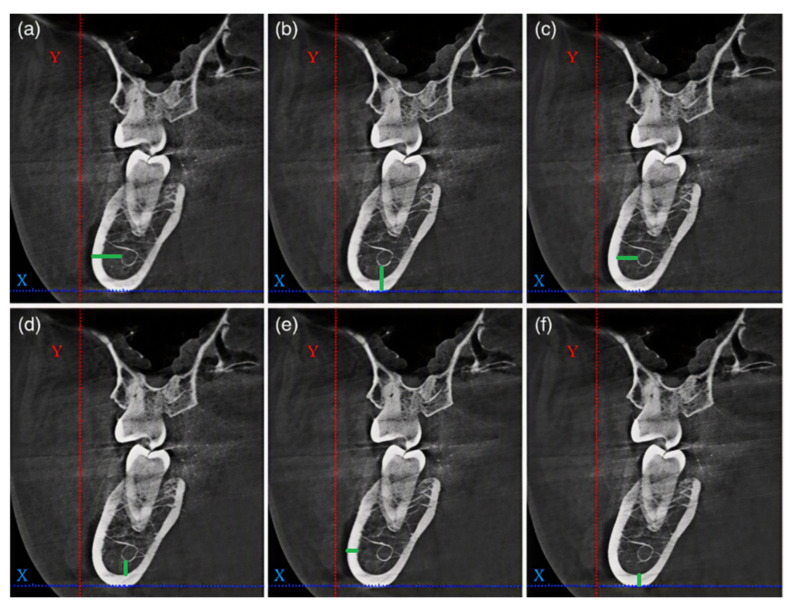
(**a**) Distance between the mandibular canal and the outer buccal cortical border (green line). (**b**) Distance between the mandibular canal and the outer inferior cortical border (green line). (**c**) Thickness of the cancellous bone located buccal to the mandibular canal (green line). (**d**) Thickness of the cancellous bone located inferior to the mandibular canal (green line). (**e**) Thickness of the cortical bone located buccal to the mandibular canal (green line). (**f**) Thickness of the cortical bone located inferior to the mandibular canal (green line).

- The distance from the outer buccal cortical border to the mandibular canal was measured perpendicular to the Y plane (Figure 3a) at the M1, M2, and M3 levels (M1buccal, M2buccal, and M3buccal).
- The distance from the outer inferior cortical border to the mandibular canal was measured perpendicular to the X plane (Figure 3b) at the M1, M2, and M3 levels (M1inferior, M2inferior, and M3inferior).
- The thickness of the cancellous bone buccal to the mandibular canal (Figure 3c) was measured at the M1, M2, and M3 levels (M1buccalcan, M2buccalcan, and M3buccalcan).
- The thickness of the cancellous bone inferior to the mandibular canal (Figure 3d) was measured at the M1, M2, and M3 levels (M1inferiorcan, M2inferiorcan, and M3inferiorcan).
- The thickness of the buccal cortical bone buccal to the mandibular canal (Figure 3e) was assessed at the M1, M2, and M3 levels (M1buccalcor, M2buccalcor, and M3buccalcor).
- The thickness of the cortical bone inferior to the mandibular canal (Figure 3f) was recorded at the M1, M2, and M3 levels (M1inferiorcor, M2inferiorcor, and M3inferiorcor).

**Figure 4:**
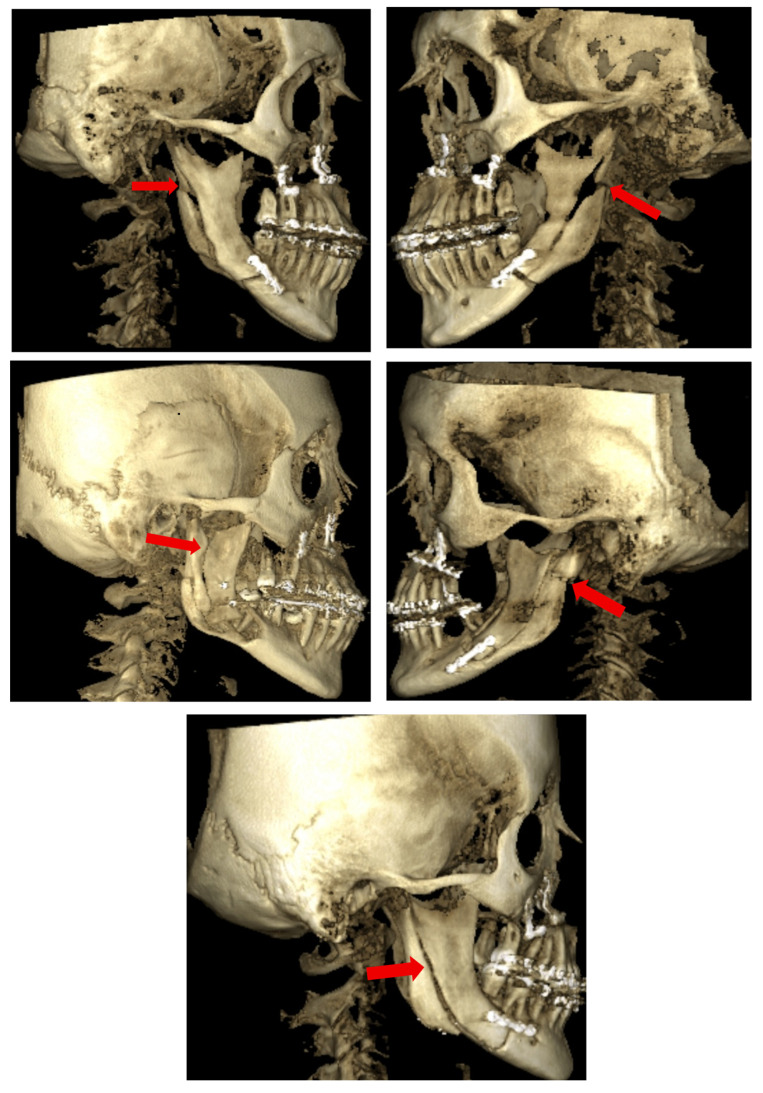

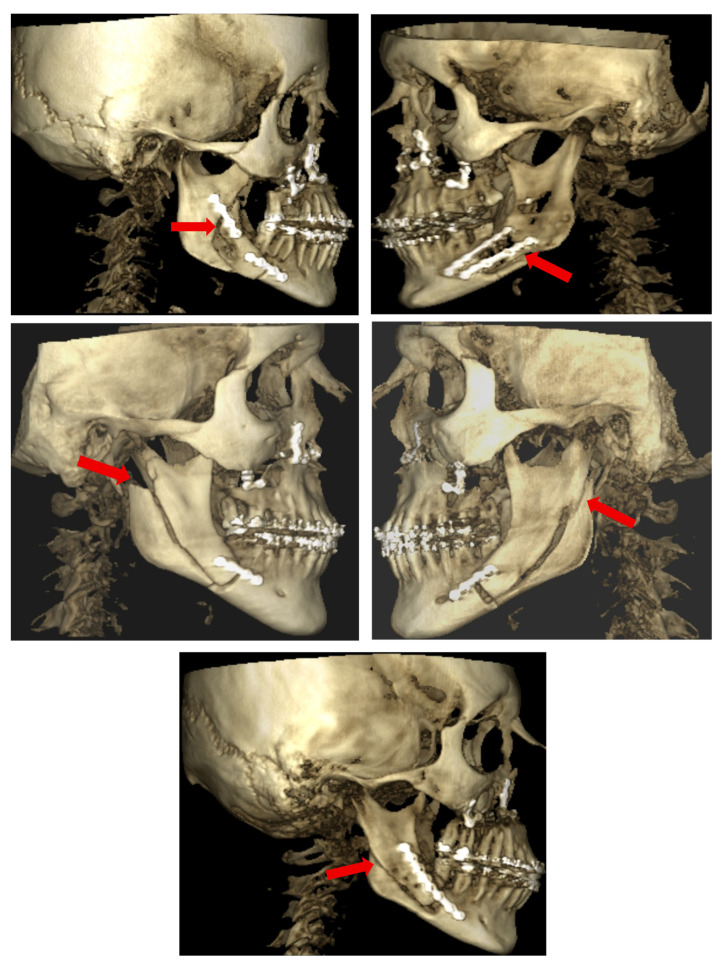
Postoperative three-dimensional CBCT reconstructions of all 10 hemimandibles with unfavorable fractures (bad splits) identified following sagittal split osteotomy. Red arrows indicate the fracture sites, illustrating the location and extent of the fracture lines in each affected hemimandible.

Each parameter measured from the right and left sides of the patient was evaluated separately. Additionally, the surgical notes and postoperatively obtained CBCT images were reviewed to check for the presence of bad splits, and any instances of bad splits were recorded in the patient data (Figure 4).

To evaluate the reliability of the measurements, intra-observer agreement was assessed by having the same researcher repeat the measurements on images from 15 randomly selected patients after a 2-month interval, with intraclass correlation coefficient (ICC) values reported. Inter-observer agreement was assessed by a second researcher performing measurements on the same images, with the ICC values also reported. Interpretation of the ICC values followed Landis and Koch’s [16] established criteria: ICC < 0.20 (poor agreement), 0.21 to 0.40 (fair agreement), 0.41 to 0.60 (moderate agreement), 0.61 to 0.80 (good agreement), and 0.81 to 1.00 (excellent agreement).

During the preparation of this manuscript, the authors used ChatGPT-4o (OpenAI, San Francisco, CA, USA) solely for English language editing, readability improvement, and grammatical refinement. The tool was not used for study design, data collection, data analysis, interpretation of results, generation of scientific content, figures, tables, or conclusions. Following the use of this tool, the authors carefully reviewed and edited all content and took full responsibility for the accuracy and integrity of the manuscript.

### 2.6. Statistical Analysis

The statistical analysis of the data was performed using the SPSS software (Statistical Package for the Social Sciences), version 25.0 (IBM Corp., Released 2010. IBM SPSS Statistics for Windows, Version 25.0. Armonk, NY, USA). Categorical variables are summarised as frequencies and percentages, whereas continuous variables are presented as the means and standard deviations (where appropriate, medians and ranges are also reported as minimum and maximum values). Fisher’s exact tests were used for the comparison of categorical variables. The Shapiro–Wilk test was used to determine whether the parameters in the study followed a normal distribution. The Mann–Whitney U test was used to analyse differences between the groups. Statistical significance was established at *p* < 0.050.

## 3. Results

The study included 112 hemimandibles from 56 patients who underwent bilateral SSO. Both the right and left sides of each patient were included in the analysis. Of these patients, 36 (64.3%) were female, and 20 (35.7%) were male. The mean age of the patients was 23.9 ± 6.1 years (range 17–46). The mean postoperative follow-up period was 33.9 ± 19.2 months (range 6–72). Regarding the underlying skeletal deformities, 10 patients (17.9%) were diagnosed with Class II malocclusion, whereas 46 patients (82.1%) presented with Class III deformities. Impacted third molars were identified in 12 patients (10.7%). A total of 10 bad splits were observed, resulting in an incidence of 8.9% per site (10/112) and 14.3% per individual (8/56). Specifically, bilateral bad splits were detected in 2 patients, while 6 patients exhibited unilateral involvement. Among the 10 bad splits, six occurred on the right side and four on the left. No significant differences were found between the two groups for sex (*p* = 0.84), age (*p* = 0.43), and side of the bad split (*p* = 0.41). Moreover, no statistically significant correlation was established between the presence of impacted third molars (*p* = 0.93), the type of preoperative skeletal deformity (*p* = 0.68), and the incidence of bad splits (Table 1). Based on the reliability of measurement assessment, ICC values showed that most parameters had good (ICC 0.61 to 0.80) to excellent (ICC 0.81 to 1.00) agreement, with no values falling below the good agreement level.

The mean distance between the inferior alveolar nerve and the inferior cortical border of the mandible on the mesial side of the second molar (M2 Inferior) was significantly greater in sides with bad splits than in those without (*p* = 0.04). No significant differences were observed between the groups in terms of the other parameters presented in Table 2A,B.

## 4. Discussion

SSO is a technically demanding procedure with potential intraoperative and postoperative complications [4]. Among the intraoperative complications, a bad split has been reported as the most common [17]. Mensink et al. [18] reported the incidence of bad splits in 17 cases (2.0%) of 851 SSO sites performed across 427 patients from 1994 to 2011. Chrcanovic and Freire-Maia [3], in their review of 21 studies, reported incidence rates per patient ranging from 0.21% to 22.72%. Balaji [19] reported bad splits in 27 cases (6.5%) of 416 SSO sites. Aarabi et al. [7] reported 14 bad splits (14.6%) among 96 SSO sites. A meta-analysis by Verweij et al. [20] included 18 retrospective and 3 prospective studies on bad splits, encompassing 8225 patients who underwent 16,359 SSO procedures, with a total of 381 bad splits, yielding an overall incidence of 2.3% per site (range: 0.5% to 14.6%).

A total of 10 bad splits were observed in the present study, resulting in an incidence of 8.9% per site and 14.3% per individual. This relatively high incidence may be explained by the nature and specific inclusion criteria of the present study, which enrolled only patients with both preoperative and postoperative CBCT scans. Postoperative CBCT imaging is not performed routinely for all orthognathic surgery patients; however, it is commonly obtained in cases where a bad split has occurred. Therefore, the inclusion of only those patients with pre- and postoperative CBCT may have led to a selective concentration of suspected or confirmed bad split cases, consequently contributing to an observed rate that is close to the upper range of results from other studies in the literature.

Many factors, including age, sex, the presence of impacted third molars, different surgical techniques and tools, surgical experience, and mandibular anatomy, may affect the risk of a bad split. A recent retrospective CBCT-based study by Suassuna et al. also supported the multifactorial nature of bad splits, emphasising that both patient-specific anatomical characteristics and technical aspects of the osteotomy may influence the occurrence of unfavorable fractures [21].

Mensink et al. [18] reported that 8 of the 17 bad splits in their study were associated with the presence of impacted third molars. However, several studies and meta-analyses have indicated that the presence of impacted third molars during surgery does not affect the incidence of bad splits [22,23,24]. Accordingly, no significant relationship was found between the presence of third molars and the occurrence of bad splits in the present study.

Many studies reported no significant relationship between age and sex and the incidence of bad splits [18,22,23,24]. Kriwalsky et al. [23], in contrast, reported an association between older age and bad splits, with an average age of 35 years for patients with bad splits compared to 25 years for those without. In the present study, no associations were found between sex or age and the occurrence of bad split.

Mensink et al. [18] and Jiang et al. [24] reported no statistically significant associations between bad split occurrence and preoperative skeletal relationship. These findings are consistent with the present study. Conversely, Kalabalık et al. [25] suggested that prognathic mandibles, characterised by reduced mediolateral width and a lower proportion of cancellous bone, may be more susceptible to bad splits. However, this conclusion was reached without statistical support. Similarly, the majority of patients in the present study had Class III skeletal deformities, and most of the bad splits occurred in Class III patients; however, this association did not reach statistical significance.

Several studies have analysed various mandibular anatomical parameters between patients with bad splits and those without during the SSO procedure [1,7,15].

Wang et al. [1] reported no significant relationship between the occurrence of bad split and various anatomical parameters, including the buccolingual thickness of the ramus at the level of the lingula and the anteroposterior length of the ramus at the same level. No association was observed with the distance from the mandibular foramen to the anterior border of the ramus, as well as the distance between the outer inferior cortical border of the mandible and the mandibular canal at the distal of the second molar. Aarabi et al. [7] reported that while the buccolingual thickness of the ramus at the level of the lingula was significantly thinner in patients with bad splits, no significant difference was observed between the groups in terms of the anteroposterior length of the ramus. Similar anatomical parameters were examined in the present study. Consistent with these findings, no correlation was found between the anteroposterior length of the ramus and bad splits. In contrast to Aarabi et al., no significant difference was observed in the buccolingual thickness of the ramus at the level of the lingula.

Telha et al. [15] found that a reduced distance between the mandibular canal and the buccal cortical border, as well as decreased cancellous bone thickness buccal to the mandibular canal, were significantly associated with an increased incidence of bad splits. In contrast, the present study found that neither of these parameters was related to bad split incidence. Additionally, Telha et al. [15] reported no association between bad splits and other anatomical parameters, such as the anteroposterior length of the ramus at the mandibular foramen, the distance from the lingula to the sigmoid notch, the mediolateral thickness of the ramus at the lingula, and cortical bone thickness buccal to the mandibular canal. In line with this, the present study demonstrated no significant correlation between these parameters and the occurrence of bad splits.

In contrast to these studies, we found that the distance between the inferior alveolar nerve and the inferior cortical border of the mandible in the region between the first and second molars was significantly greater in the bad split group. Song and Kim reported that the risk of bad splits increases when the inferior border osteotomy does not extend fully through the caudal cortex to the lingual cortex [26]. Although the extent and completeness of the inferior border osteotomy were not quantitatively assessed in the present study, a greater distance between the mandibular canal and the inferior cortical border may require a deeper inferior border osteotomy to achieve complete cortical separation. Interestingly, when evaluated separately, neither the cortical bone thickness nor the cancellous bone thickness inferior to the mandibular canal differed significantly between the groups. However, the total distance between the mandibular canal and the inferior cortical border was significantly greater in the bad split group. We hypothesise that while the increased amount of cancellous bone could have led the surgeon to perceive the osteotomy as complete, the persisting cortical integrity at the inferior border may have resulted in an unfavorable transfer of forces toward weaker areas of bone during splitting. This suggests that the combined anatomical relationship may be of greater clinical significance than either component considered individually. Therefore, our findings should be interpreted with caution, as technical variations in the inferior border osteotomy may also have contributed to the occurrence of bad splits.

On the other hand, Houppermans et al. [27] reported that an inferior border cut extending through the lingual cortex does not necessarily result in greater predictability of a split in SSO. Also, several studies have reported that extension of the inferior border osteotomy toward the lingual cortex may increase the risk of postoperative inferior border defects in mandibular advancements [28,29,30].

Taken together, these findings suggest that achieving complete cortical separation may be important for reducing the risk of bad splits, while excessive extension of the inferior border osteotomy should also be avoided because of its potential adverse effects.

This study has several limitations. First, its retrospective and single-centre design inherently restricts the external validity and generalizability of the findings. Moreover, as the surgical procedures were performed by multiple operators, a comprehensive evaluation of the potential effect of surgeon experience on clinical outcomes was not feasible. Furthermore, routine postoperative CBCT imaging was not systematically performed in all orthognathic surgery patients. From these limitations, several conclusions can be drawn. Age, sex, presence of third molars, and type of deformity were not found to be associated with an increased number of bad fractures. However, the average bone thickness between the mandibular canal and the inferior cortical border in the region between the first and second molars was greater in the bad split group. We suggest that increased bone thickness at the inferior border may lead to incomplete osteotomy in some cases, resulting in reduced structural integrity of the proximal segment during the separation process, and thus may contribute to bad splits. We believe that CBCT assessment of mandibular anatomy in orthognathic surgery patients may help reduce the incidence of bad splits. Furthermore, future prospective studies are needed in which both preoperative and postoperative CBCT assessments are performed in all patients, and the precise location of osteotomies is determined using these images.

## Figures and Tables

**Table 1:** Differences in Demographic and Clinical Characteristics Between Bad Split and Non-Bad Split Groups.

	Without Bad Split	With Bad Split	*p* ^†^
	*n* (%)	*n* (%)	
**Sex**			
Female	31 (55.3)	5 (8.9)	0.84
Male	17 (30.3)	3 (5.3)	
**Side**			
Right	50 (89.3)	6 (10.7)	0.41
Left	52 (92.9)	4 (7.1)	
**Deformity**			
Class II	9 (16.0)	1 (1.8)	0.68
Class III	39 (69.6)	7 (12.5)	
**Impacted Third Molar**	11 (10.8)	1 (10)	0.93
	**Med (IQR)**	**Med (IQR)**	***p*** ^‡^
Age	22 (6)	23 (5)	0.43

Med: Median, IQR: Interquartile Range, ^†^: Fisher’s exact test, ^‡^: Mann–Whitney U test.

**Table 2:** Differences Between Morphological Evaluation Findings on Preoperative CBCT and Bad Split Groups.

	Without Bad Split (*n* = 102)	With Bad Split (*n* = 10)	*p* ^‡^
	Med (IQR)	Med (IQR)	
(**A**)			
M2toFor	10.0 (4.61)	8.81 (7.72)	0.55
LintoSig	16.82 (4.23)	15.20 (6.67)	0.31
RanttoFor	12.53 (2.84)	11.57 (3.02)	0.24
Rammedlat	4.82 (1.98)	4.81 (2.12)	0.39
RanttPost	28.96 (3.93)	29.49 (5.13)	0.98
M1 buccal	4.47 (1.31)	4.6 (2.74)	0.90
M2 buccal	5.20 (1.69)	5.20 (2.69)	0.99
M3 buccal	4.40 (2.00)	4.80 (2.50)	0.62
M1 inferior	5.88 (2.21)	6.38 (1.72)	0.22
M2 inferior	5.60 (2.22)	6.76 (1.65)	0.04 *
M3 inferior	6.03 (2.39)	7.00 (3.22)	0.13
(**B**)			
M1 buccalcan	2 (1.56)	2.20 (2.80)	0.79
M2 buccalcan	2.42 (1.59)	2.60 (2.19)	0.89
M3 buccalcan	2.00 (2.01)	2.00 (2.07)	0.45
M1 inferiorcan	2.47 (1.68)	3.05 (1.16)	0.19
M2 inferiorcan	2.04 (2.08)	2.66 (0.97)	0.11
M3 inferiorcan	2.97 (2.47)	3.31 (2.91)	0.36
M1 buccalcor	2.43 (0.43)	2.48 (0.50)	0.78
M2 buccalcor	2.80 (0.45)	2.74 (0.50)	0.64
M3 buccalcor	2.80 (0.80)	2.80 (0.80)	0.88
M1 inferiorcor	3.30 (0.86)	3.80 (0.74)	0.93
M2 inferiorcor	3.51 (0.79)	3.60 (0.78)	0.09
M3 inferiorcor	3.20 (0.62)	3.21 (0.93)	0.10

Med: Median, IQR: Interquartile Range, * *p* < 0.050, ^‡^: Mann–Whitney U test.

## Data Availability

The datasets generated and/or analysed during the current study are not publicly available owing to patient confidentiality and institutional regulations but are available from the corresponding author upon reasonable request.
